# Association Between Subnational Vaccine Coverage, Migration, and Incident Cases of Measles, Mumps, and Rubella in Iraq, 2001–2016

**DOI:** 10.3389/fpubh.2021.689458

**Published:** 2022-01-20

**Authors:** Haley Comfort, Riyadh K. Lafta, Abraham D. Flaxman, Amy Hagopian, Herbert C. Duber

**Affiliations:** ^1^Department of Global Health, University of Washington, Seattle, WA, United States; ^2^Institute for Health Metrics and Evaluation, University of Washington, Seattle, WA, United States; ^3^Department of Family and Community Medicine, Al Mustansiriya University, Baghdad, Iraq; ^4^Department of Health Metrics Sciences, University of Washington, Seattle, WA, United States; ^5^Department of Health Systems and Population Health, University of Washington, Seattle, WA, United States; ^6^Department of Emergency Medicine, University of Washington, Seattle, WA, United States

**Keywords:** vaccination, conflict, measles, Iraq, MMR

## Abstract

**Objective:**

This analysis examines governorate-level disease incidence as well as the relationship between incidence and the number of persons of concern for three vaccine-preventable diseases—measles, mumps, and rubella—between 2001 and 2016.

**Methods:**

Using Iraqi Ministry of Health and United Nations High Commissioner for Refugees (UNHCR) data, we performed descriptive analyses of disease incidence and conducted a pooled statistical analysis with a linear mixed effects regression model to examine the role of vaccine coverage and migration of persons of concern on subnational disease incidence.

**Results:**

We found large variability in governorate-level incidence, particularly for measles (on the order of 100x). We identified decreases in incident measles cases per 100,000 persons for each additional percent vaccinated (0.82, 95% CI: [0.64, 1.00], *p*-value < 0.001) and for every additional 10,000 persons of concern when incorporating displacement into our model (0.26, 95% CI: [0.22, 0.30], *p*-value < 0.001). These relationships were insignificant for mumps and rubella.

**Conclusions:**

National level summary statistics do not adequately capture the high geospatial disparity in disease incidence between 2001 and 2016. This variability is complicated by MMR vaccine coverage and the migration of “persons of concern” (refugees) during conflict. We found that even when vaccine coverage was constant, measles incidence was higher in locations with more displaced persons, suggesting conflict fueled the epidemic in ways that vaccine coverage could not control.

## Introduction

Throughout the 1970s and 1980s, the health system in Iraq was well-regarded in the region and able to meet the health needs of Iraqis (1). However, momentum dissipated in the following years as leadership struggles, the Gulf War, and sanctions redirected health resources and weakened infrastructure (1). The United States-led invasion of Iraq began on March 20, 2003, peaked in 2006, and was followed by a formal occupation through the year 2011 (2). In the years following the formal end of the U.S. occupation, armed conflict continued. The extremist group, Islamic State (ISIS), carried out attacks beginning in 2011 but became a serious problem in 2014 when it launched larger attacks on Iraqi cities like Mosul and Tikrit, expanding territory under its control (3). Throughout the next three years, civil unrest ensued, although ISIS lost control of 95% of its territory by the end of 2017 (3).

One of the key activities that a health system provides is the provision of childhood immunizations. A strong vaccine delivery system requires a well-described chain of events concerning storage, handling, and stock management (4). To ensure the uninterrupted availability of quality vaccines, logistics management systems address rigorous temperature control and information systems; a primary goal is to ensure opportunities to vaccinate are not missed because vaccines are unavailable (4). Not surprisingly, conflict drastically undermines supply chains and access to services, and within a few months of the initial invasion, an estimated 210,000 infants were unable to receive necessary vaccinations after birth (5).

The United Nations International Children's Emergency Fund estimated that in 2017, about two-thirds of the 20.8 million children without a single dose of a measles vaccine were in areas affected by conflict (6–8). However, despite conflict and insecurity since the 2003 invasion, Iraq experienced only about 0.12 attacks on health facilities per month between 2003 and 2011, a much smaller average than in other conflict-ridden countries (9). Although early impacts on the national vaccination program were significant, the Iraqi Ministry of Health (MoH) and humanitarian groups were largely successful in maintaining vaccine operations, especially for children (4, 10, 11). This includes the coordination of vaccination campaigns to administer the measles, mumps, and rubella (MMR) vaccine to vulnerable populations nationwide, including hard to reach locations (12). The National Immunization schedule recommends children receive the measles vaccine at nine months and MMR vaccines at 15 months and between 4 and 6 years (13).

A recent analysis of communicable disease incidence by Zhao et al., using the same dataset as this paper, provides insights into national trends of vaccine-preventable disease in the post-invasion years (14). Outbreaks of measles, mumps, and rubella were observed, with 30,321 measles cases in 2009, 12,815 and 74,212 mumps cases in 2004 and 2016, respectively, and 290 rubella cases in 2004 (14). Further analyses performed by Lafta and Hussain examined the relationship between national vaccine coverage and outbreaks of vaccine-preventable illness, finding that fluctuations in vaccine coverage precipitated outbreaks (15).

However, these papers offer national trends alone, covering over geospatial variation essential for identifying and explaining outbreaks. The World Health Organization recommends subnational situation analysis to inform policy design, priority-setting, and the allocation of resources (16). In Iraq, conflict was not evenly distributed; areas with more conflict experienced more outmigration as those in harm's way sought refuge in safer regions (17). Subnational analyses can help predict factors leading to outbreaks (16). Public health planning and implementation of new programs can then address the local concerns.

In this study, we examine a time series of subnational Iraq disease incidence for measles, mumps, and rubella, from 2001 to 2016. We identify outbreak locations and assess the role of subnational vaccine coverage and population change on disease incidence. Finally, we take a closer look at “persons of concern” (internally displaced persons and refugee migrants, mostly), to see if the presence of these highly vulnerable individuals is related to change in disease incidence.

## Materials and Methods

Our study covered Iraq's population, estimated to include about 26.41 million persons in 2000, 34.36 million persons in 2010, and 43.30 million in 2017 (18).

### Data Sources

The primary dataset for this analysis comes from the Iraqi Ministry of Health (MoH). The dataset contains records at the governorate level for 32 communicable diseases across four broad categories: (1) vaccine-preventable diseases, (2) other World Health Organization (WHO) surveillance diseases, (3) other vector-borne and zoonotic diseases, and (4) reemerging diseases (14). Per Iraqi MoH policy, all public-sector hospitals and primary health care centers report their monthly case totals of vaccine-preventable diseases to the Preventive Health Department at the Directorate General of Health for their respective governorate. The information is then reported to the Communicable Disease Control Unit of the Ministry of Health in Iraq the first week of each month for the prior month's totals.

The dataset also includes annual estimates of governorate-level population, case counts, and vaccine coverage. Coverage is defined as the number of persons vaccinated divided by target population and is considered "complete” upon receipt of two doses of the vaccine, generally administered at 15 months and 5 years of age. Persons of concern are included in these coverage estimates, and campaign doses are not included.

We also used a supplemental dataset from The United Nations High Commissioner for Refugees (UNHCR) entitled *Demographics for UNHCR's populations of concern residing in Iraq* (17). UNHCR defines “persons of concern” as refugees (people fleeing conflict or persecution from another country), returnees, stateless people, internally displaced Iraqis, and asylum seekers (19). This dataset, available from 2005 to 2016, reports annual estimates of the number of “person of concern” in various areas across Iraq. We calculated sex-specific totals for each governorate by year from 2005 to 2016 by aggregating all locations to the corresponding governorate. About 120,000 persons of concern, accounting for about 2.5% of the total, had to be removed from the dataset due to unknown locations or their assignment to “No Man's Land” (unoccupied or disputed land).

### Analysis

We analyzed data from the vaccine-preventable diseases category of the MoH dataset, specifically focusing on case counts of three preventable diseases—measles, mumps, and rubella—from 2001 to 2016. We calculated annual disease incidence at the governorate level per 100,000 persons using the equation:


$$\begin{array}{l} incidence_{year}=\;\frac{{\#\;cases}_{year}}{population_{year}}\;\times \;100,000 \end{array}$$

This was descriptively analyzed as a geospatial time series. To visually examine the geospatial distribution of disease, the Iraq MoH dataset was merged with shapefiles of the administrative level 1 (governorate-level) boundaries acquired from the Humanitarian Data Exchange website (20).

For our descriptive analysis, we transformed absolute governorate-level population counts from the Iraq MoH dataset into population density for each governorate as well as annual percent change in population. Population change over time identified the periods when governorates experienced a stable population vs. time periods with significant movement into or out of the governorate.

We then performed a regression analysis to produce a pooled estimate of the risk difference for the relationship of interest between disease incidence and vaccine coverage. In our linear mixed effects regression model, we set vaccine coverage as the independent variable and disease incidence per 100,000 as the dependent variable. We also included governorate as a random effect term to control for any correlation in incidence within governorates. For this analysis, we offset the vaccine coverage variable by two years prior to account for outbreaks that started and rolled into the year reporting the main outbreak. In equations, our model took the form


$$\begin{array}{l} incidence_{gov,year}\;\sim \;coverage_{gov,year-2}+u_{gov}, \end{array}$$

where gov denotes the governorate, year the calendar year, and *u*_*gov*_ is the governorate-level random effect.

Finally, to identify whether an interaction between vaccine coverage and the number of persons of concern existed, we ran an additional version of our pooled linear mixed effects regression model which included an interaction term for this relationship of interest. For this model, we used a subsetted version of our data that started in 2005 and included only the governorates and years when governorates reportedly hosted persons of concern.

All data cleaning, management, and analysis was done using R (version 3.5.0). Maps were created using the *ggplot2* package.

### Ethical Approval

The use of de-identified aggregate routine health surveillance data on the study population did not require Institutional Review Board approval, as deemed by the Human Subjects Division of the University of Washington.

## Results

### Incidence of Measles, Mumps, and Rubella

**Figure 1:**
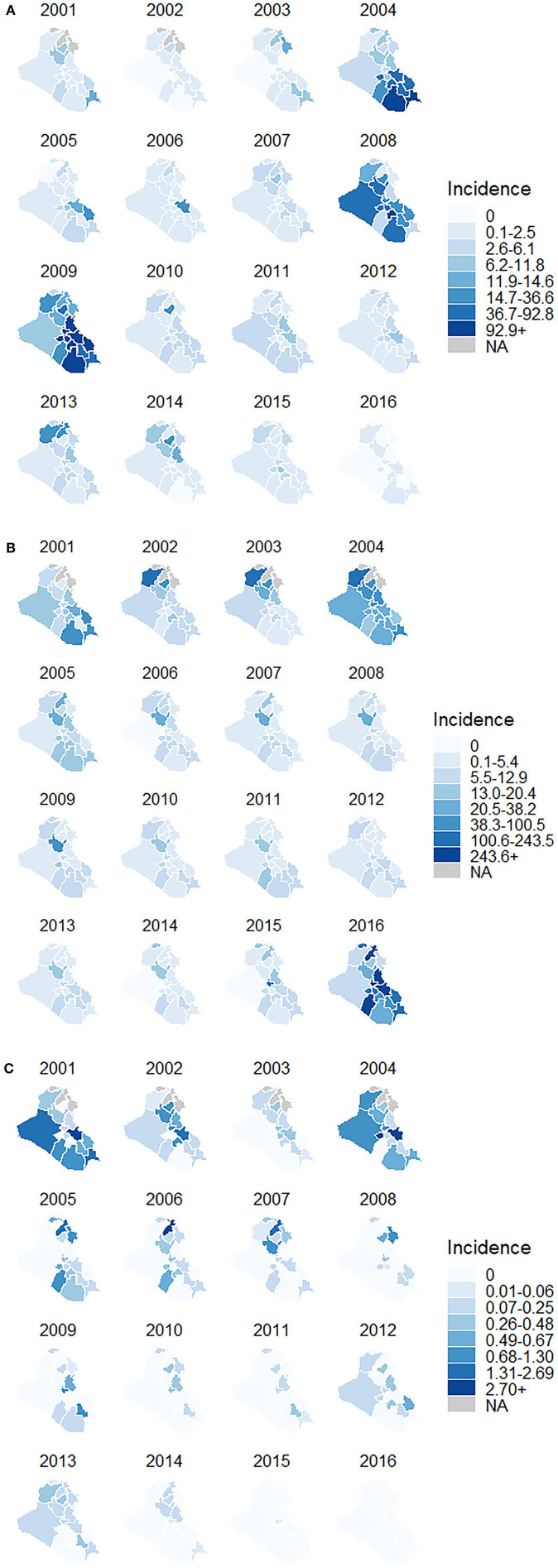
**(A)** Measles incidence per 100,000 persons in Iraq by governorate, 2001–2016 **(B)** mumps incidence per 100,000 persons in Iraq by governorate, 2001–2016 **(C)** rubella incidence per 100,000 persons in Iraq by governorate, 2001–2016.

Figure 1 reports measles, mumps, and rubella incidence per 100,000 persons by governorate, demonstrating the remarkable uptick in cases during outbreaks. In 2008–2009, Iraq experienced a major measles outbreak, with 30,321 reported cases in 2009 alone (21). During the outbreak, 11 of the 18 governorates reported their highest incidence of the time-series. While 10 governorates reported incidences near or fewer than 60 cases per 100,000 persons, eight governorates reported incidences larger than 120 per 100,000. Dahuk, the northwestern most governorate, reported the lowest incidence of 3.9 per 100,000 and Wassit, located southeast of Baghdad, reported the highest incidence of 379.3 per 100,000. A smaller outbreak of 9,081 cases was also identified in 2004, primarily in the southernmost governorates. Four governorates—Dahuk, Basrah, Muthanna, and Thi-Qar—reported this year as their highest incidence.

Two major outbreaks of mumps occurred between 2001 and 2016 in Iraq, with more than 10,000 cases reported in each incident. The first outbreak was in 2004, and a second, larger episode, extended from 2015 to 2016. In 2004, the governorate of Baghdad saw the most cases of all governorates in that year (3,768 cases). The outbreak spread slightly, with additional cases reported mostly in governorates north of Baghdad. In 2015, the year preceding the second major outbreak, Baghdad governorate saw 10,794 cases and then about four times that number in 2016. The outbreak in 2016 resulted in 13 of the 18 governorates reporting their highest incidence of mumps in the time-series.

Iraq had one outbreak of rubella in 2004. During this outbreak, Kerbala, located southwest of Baghdad, reported the highest incidence of rubella (14.4 per 100,000 persons) and the largest number of cases (113 total). In other years, the governorate of Baghdad often reported the greatest number of cases per year, but because of its larger population, overall incidence stayed relatively low and was comparable to that of other governorates. While several other smaller outbreaks did occur, the disease had limited spread to neighboring governorates, maintaining a relatively stable incidence of fewer than one per 100,000 persons per year, likely due to the low transmissibility of rubella compared to the other infectious diseases we examined.

### Vaccine Coverage and Disease Incidence

**Figure 2:**
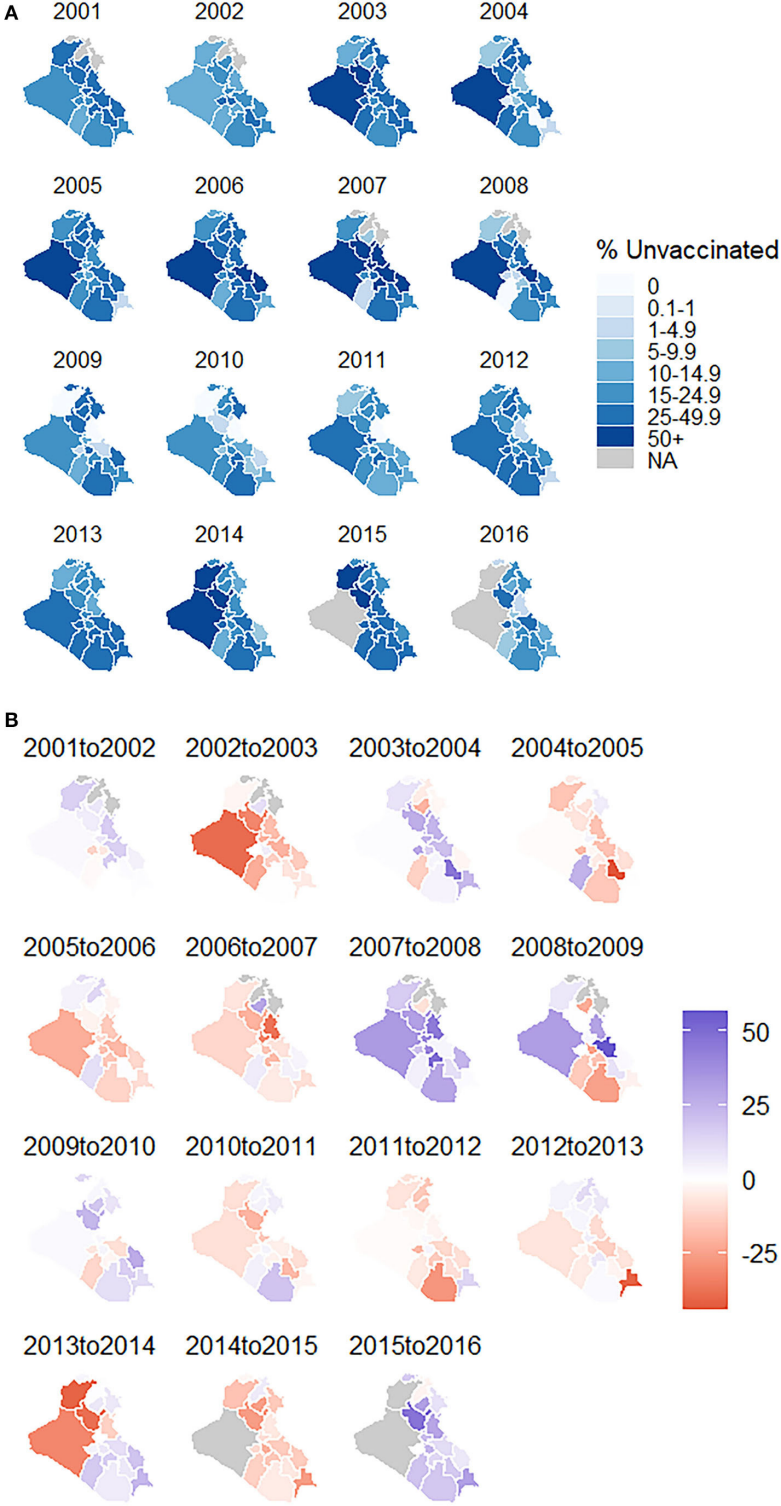
**(A)** MMR vaccine coverage in Iraq by governorate, 2001–2016 **(B)** absolute change in MMR vaccine coverage from previous year in Iraq by governorate, 2001–2016.

We found significant governorate-level disparities in MMR vaccine coverage, shown in Figure 2. The percent of vaccinated children younger than 5 years at the governorate level ranged from 14% (Anbar in 2007) to 100% (Ninewa from 2009 to 2010, Diyala from 2009 to 2011, Najaf in 2008, and Thi-Qar in 2004). The largest increase in vaccine coverage, a 57% absolute change, occurred between the years 2008 and 2009 in the eastern governorate of Wassit. The largest decrease in vaccine coverage, a 44% absolute change, occurred between the years 2004 and 2005 in the southern governorate of Thi-Qar.

The linear mixed effects model identified a positive, significant association between annual measles incidence per 100,000 persons and MMR vaccine coverage two years prior. For each additional percent vaccinated, the risk difference in the measles incidence rate was 0.82 cases per 100,000 persons lower (95% CI: [0.64, 1.00], *p*-value < 0.001), controlling for governorate.

The models did not find significant associations between MMR vaccine coverage and incidence of mumps or incidence of rubella. The models suggested mumps incidence decreased with decreasing vaccine coverage and there was almost no difference in rubella incidence as vaccine coverage increased.

### Migration, Vulnerability, and Disease Incidence

Between 2001 and 2007, the total population in each governorate remained relatively consistent with the annual increase fluctuating between 2.5 and 3.3%. The only governorate to stand out from this pattern, Salah al-Din in central Iraq, reported decreases in population of 1.3% from 2005 to 2006 and 21.4% from 2006 to 2007, following a mosque-bombing and subsequent violence in Samarra, the governorate's most populated city (22).

The first major governorate-level population shift in the post-invasion era occurred between 2007 and 2008, when 14 out of the 18 governorates experienced greater than a 5% increase or 5% decrease in population. During this time, it appears many Iraqis moved from Diyala and Sulaymaniyah in northeastern Iraq toward the neighboring westerly governorates of Erbil, Kirkuk, and Salah al-Din. UNHCR identified between 40,000 to 50,000 persons of concern in Iraq for each year between 2005 and 2008, but this number spiked to more than 575,000 in 2009. From 2009 to 2010, we observed a reversal compared to 2007 and 2008 when 12 governorates experienced a population change in the opposite direction.

Large-scale displacement and migration continued between 2010 and 2011. Sixteen governorates experienced either increases or decreases in population in the same direction as what had been observed between 2007 and 2008. Interestingly, the six governorates with the greatest percent increases in population between 2009 and 2010 had the greatest percent decreases between 2010 and 2011. In 2010, UNHCR estimates more than 1.5 million persons of concern were dispersed throughout the country. The governorate of Baghdad had the largest number of persons of concern, with 428,647 arrivals. Two other governorates, Ninewa and Diyala, hosted at least 100,000 persons of concern in 2010. While displacement dropped in 2011, more than 850,000 persons of concern were still displaced across Iraq in 2012.

**Figure 3:**
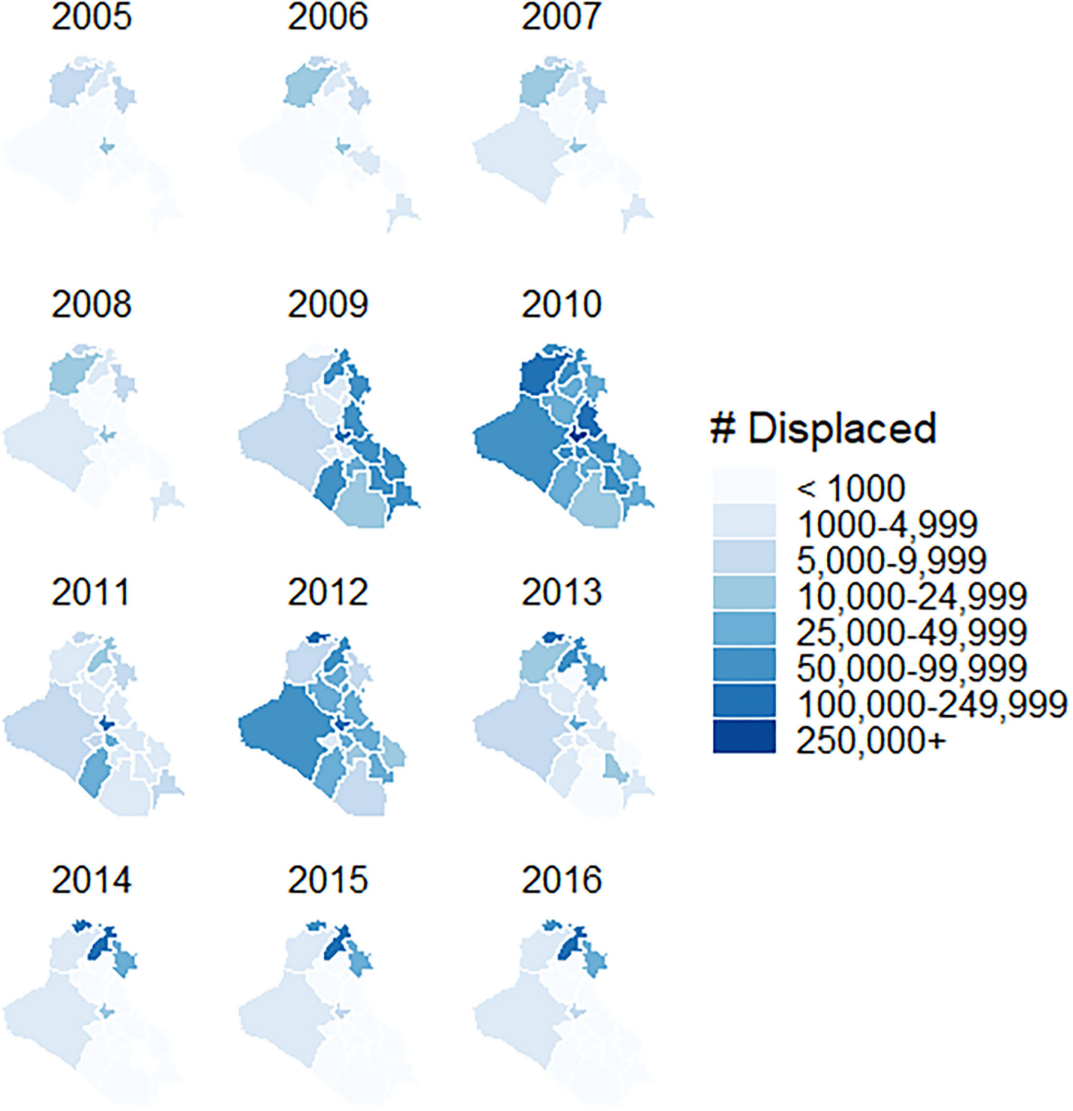
Total number of persons of concern in Iraq by governorate, 2001–2016.

After these large redistributions of the population during the peak of the war, governorates returned to their pre-2007 patterns, with almost all governorates experiencing a 2 to 3% increase in population per year. From 2014 to 2016, the three northernmost governorates, making up most of the Kurdistan region, hosted the greatest number of persons of concern. These migration trends over time are shown in Figure 3.

Our regression analysis identified a significant relationship between persons of concern and MMR vaccine coverage for measles. For every additional 10,000 persons of concern residing within a governorate, the difference in measles incidence was 0.26 cases per 100,000 lower (95% CI: [0.22, 0.30], *p*-value < 0.001) among groups that differ by one percentage point in vaccine coverage. For mumps, the difference in incidence was 0.02 cases per 100,000 higher (95% CI: [-0.05, 0.08], *p*-value = 0.763), but this value was not statistically significant. There was no relationship between the number of persons of concern in a governorate and MMR vaccine coverage for rubella.

## Discussion

Between 2007 and 2011, most governorates experienced major shifts in population, as Iraqis became internally displaced within their home governorate or another governorate. This was further complicated by conflict in Syria and the movement of Syrian refugees into Iraq (23). Many relocated, often without an option to return, due to threats, lack of security, and fear of violence (24). This created pockets of individuals vulnerable to vaccine-preventable diseases within governorates, and it led to a diagnosed case of polio in Iraq for the first time in 14 years (25).

Vaccine campaigns are a routine WHO response in war and conflict, including in Iraq. Several vaccination campaigns protected vulnerable populations against measles, mumps, and rubella and prevented additional transmission when nearby individuals did become infected. Some of the major post-invasion campaigns took place across Iraq in 2003 and 2007 (11, 26). Other campaigns in 2008 and 2009 targeted specific areas to limit the spread of current outbreaks (21, 27, 28). These campaigns improved levels of coverage and herd immunity which likely helped mitigate observed outbreaks and prevent additional outbreaks from occurring.

Lafta and Hussain's analysis reported an average measles incidence of 2.6 per 100,000 persons when excluding the outbreak years of 2004, 2008–2009, and 2014 and an incidence of 95.7 per 100,000 in 2009 (15). However, this national incidence figure masked the significant geographic disparity in incidence among the governorates. We identified large ranges in incidence of measles, mumps, and rubella, especially during the outbreak years. Further, our analysis using subnational-level data revealed disproportionately higher numbers of cases and subsequent outbreaks in some areas and our pooled analysis indicated a relationship between decreasing vaccine coverage and increasing disease incidence. This suggests that a subnational analysis may be able to identify more and less vulnerable governorates based on factors such as vaccine coverage and the presence of higher concentrations of persons of concern.

At the same time, we find that the relationship between outbreaks of disease and baseline governorate characteristics is complex. Our findings regarding the relationship between disease incidence and various governorate factors such as vaccine coverage and persons of concerns did not hold for all diseases we examined. This does not mean these relationships do not exist, but rather there are likely many additional factors that contribute to the spread of these diseases and ultimately determine the size of an outbreak. The highly infectious nature of measles, for example, likely contributed to why a relationship was identified between vaccine coverage and incidence, unlike the other two diseases we examined. Additional factors include, but are not limited to, environmental-related factors like migration, population density, access to care, and important disease-related factors such as transmissibility, length of the infectious period, and number of contacts. Additional research can help us understand the relative importance of these variables in developing targeted programs to prevent and mitigate outbreaks in future conflicts.

Demonstrating how conflict undermines a country's health care system can provide evidence to build commitment to reduce conflict and ensure proactive response when it does occur. For the years 2001 to 2016 in Iraq, we did not identify a singular factor that predicted outbreaks of infectious disease. This is important to consider for public health planning because multiple factors, beyond just vaccine coverage and persons of concern, must be addressed to fully limit disease spread. Well-functioning health systems and targeted interventions programs are necessary to protect vulnerable individuals from preventable infectious diseases.

### Limitations

A major limitation of this data is the inability to verify its accuracy. Governorate-level case counts for measles, mumps, and rubella in the dataset are likely to represent underestimates, with the amount of bias varying by governorate. A considerable number of Iraqis who contracted an infectious disease may have been unable to access medical services in conflict areas. Since primary health centers are responsible for reporting the number of cases of notifiable diseases they diagnose, missing cases results in underreporting to the Iraq MoH. Also, health centers in the private sector do not have the same requirement to report cases. However, it is possible only a few cases were missed for this reason because private hospitals generally focus more on surgery and deliveries than on hospital care (1).

Governorate-level incidence per 100,000 persons would be underestimated if the health care system missed reporting cases of disease. It is important to consider a potential for collinearity between high conflict and low case reporting that may also be associated with low or declining vaccine coverage which could have been over or under-reported as well. It is likely that the interaction of several covariates, beyond what is included in our analysis, play into whether an outbreak occurs.

Missing data was also a problem for several governorate-years. Primarily, data was missing from the Kurdistan region of Iraq which is made up of Iraq's three northernmost governorates of Erbil, Dahuk, and Sulaymaniyah. The autonomous region now also includes Halabja Governorate which split off from Sulaymaniyah in 2014, but the dataset did not specifically represent this new governorate on its own (29).

## Conclusion

The Iraq war undermined the nation's health system, generating large fluctuations in governorate-level population and vaccine coverage, fueling outbreaks of measles and mumps. Our analysis found that higher governorate vaccine coverage was associated with lower measles incidence. Additionally, measles incidence was found to be higher in governorates with higher concentrations of persons of concern. Effective vaccination campaigns by the Iraqi Ministry of Health and partnering humanitarian organizations no doubt alleviated even larger measles outbreaks. Future conflicts will almost certainly face similar challenges, and policies that focus interventions in areas of low vaccine coverage and population movement within country will be essential to minimize the impact of vaccine preventable diseases.

## Data Availability Statement

Publicly available datasets were analyzed in this study. This data can be found here: https://zenodo.org/record/4649509.

## Author Contributions

HC, AH, and RL conceived the initial study. The analytic plan was developed by HC, AF, AH, and HCD. Data were obtained by RL and the statistical analysis was performed by HC. All authors interpreted findings. HC developed the first draft of the manuscript and all authors provided critical feedback and revision. All authors approve of the final manuscript.

## Conflict of Interest

The authors declare that the research was conducted in the absence of any commercial or financial relationships that could be construed as a potential conflict of interest.

## Publisher's Note

All claims expressed in this article are solely those of the authors and do not necessarily represent those of their affiliated organizations, or those of the publisher, the editors and the reviewers. Any product that may be evaluated in this article, or claim that may be made by its manufacturer, is not guaranteed or endorsed by the publisher.
